# The successful detection of postpartum unruptured vaginal pseudoaneurysm using ultrasonography: a case report

**DOI:** 10.1186/2193-1801-3-482

**Published:** 2014-08-28

**Authors:** Saki Gondo, Daichi Urushiyama, Toshiyuki Yoshizato, Shinichi Kora, Miyako Maehara, Haruhiko Kondo, Shingo Miyamoto

**Affiliations:** Department of Obstetrics and Gynecology, Faculty of Medicine, Fukuoka University, Fukuoka, Japan; Center for Maternal, Fetal and Neonatal Medicine, Fukuoka University Hospital, 7-45-1 Nanakuma, Jonan-ku, Fukuoka, 814-0180 Japan; Department of Radiology, Faculty of Medicine, Fukuoka University, Fukuoka, Japan

**Keywords:** Pseudoaneurysm, Vagina, Puerperium, Transvaginal ultrasonography, Contrast-enhanced computed tomography

## Abstract

**Introduction:**

We report the first case of the successful detection of postpartum unruptured vaginal pseudoaneurysm using power- and pulsed-Doppler ultrasonography after delivery.

**Case description:**

A 38-year-old primiparous Japanese woman had a vaginal laceration with pulsatile bleeding after delivering by vacuum extraction. Transvaginal ultrasonography of the repaired vaginal wall showed an 18 × 20 mm hematoma within which a 6 × 7 mm pulsating anechoic mass was detected. Power-Doppler ultrasonography showed blood flow signals in the anechoic mass. Arterial waveforms detected in pulsed-Doppler mode were suggestive of unruptured pseudoaneurysm. Careful monitoring with contrast-enhanced computed tomography showed an increase in the size of the pseudoaneurysm on the fourth postpartum day. On the sixth postpartum day, massive vaginal bleeding occurred. Emergency angiography revealed strong staining with extravasation from the left vaginal artery, confirming the diagnosis of pseudoaneurysm. Embolization for hemostasis was successfully performed.

**Discussion and evaluation:**

As far as we know, our case is the first in which an unruptured vaginal pseudoaneurysm was diagnosed using ultrasonography. The differential diagnoses of pseudoaneurysm are arteriovenous malformations including arteriovenous fistula. This case had the typical ultrasonographic patterns of pseudoaneurysm in which the presence of one or two cystic masses in B-mode and color- and/or power-Doppler flow signals was demonstrated along with high-resistance arterial flow waveforms in pulsed-Doppler mode. Sequential examinations of contrast-enhanced CT showed ongoing development of the pseudoaneurysm. In retrospect, we could have performed angiography for embolization when the unruptured pseudoaneurysm was diagnosed, or at the latest when ongoing development of the pseudo-aneurysm was recognized, irrespective of whether symptoms were present.

**Conclusions:**

Ultrasonography is a non-invasive and clinically useful modality in the differential diagnosis of pseudoaneurysm. Contrast-enhanced computed tomography with or without ultrasonography can be useful for sequential monitoring of the size of unruptured pseudoaneurysms.

## Introduction

Pseudoaneurysms arise from disruption of arterial wall continuity by inflammation or events such as vascular trauma. The pseudoaneurysm margins are formed by the thrombus originating from the injured artery and are not surrounded by three arterial layers. Pseudoaneurysm rupture can lead to devastating patient outcomes, including life-threatening bleeding. Ultrasonography is a useful modality in the diagnosis of unruptured pseudoaneurysm occurring after cesarean section or D&C (Pelage et al. 1999; Kwon and Kim 2002; Cooper et al. 2004; Kovo et al. 2007; Bouchet et al. 2012; Kim et al. 2008; Padavala and Ahluwalia 2004). Rupture of vaginal pseudoaneurysm is a rare cause of postpartum hemorrhage after transvaginal delivery; however, when it occurs, the condition can be life threatening.

There have been several reports of ruptured vaginal pseudoaneurysms diagnosed because of massive bleeding (Matsuhashi et al. 2001; Soyer et al. 2008; Arab and Dy 2011; Nagayama et al. 2011). We report the first case of the successful detection of postpartum unruptured vaginal pseudoaneurysm using power- and pulsed-Doppler ultrasonography. Sequential monitoring of the size of the pseudoaneurysm with contrast-enhanced computed tomography (CT) and ultrasonography made it possible to avoid life-threatening bleeding.

## Case report

**Figure 1:**
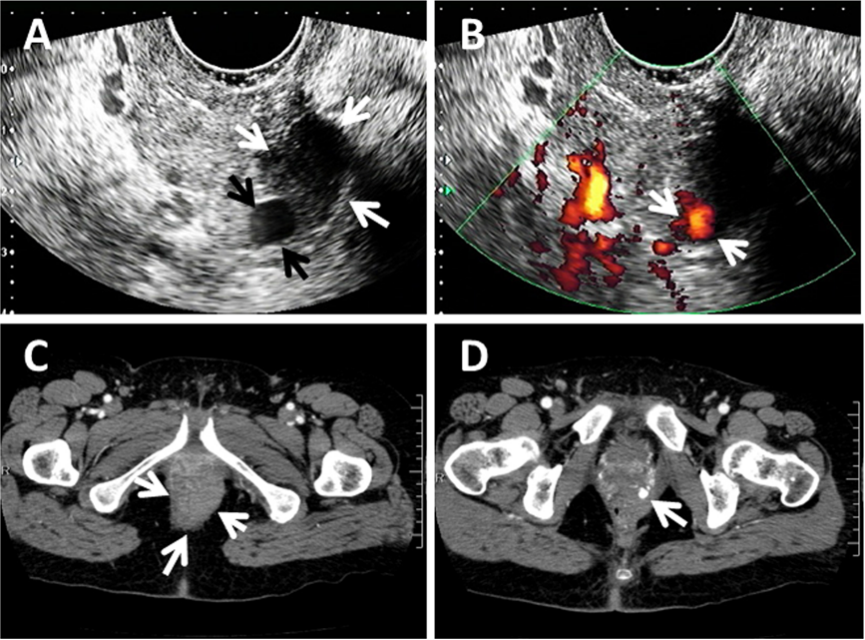
**Transvaginal ultrasonography and pelvic contrast-enhanced computed tomography on the day of delivery. (A)** pseudoaneurysm (black arrows) and adjacent hematoma (white arrows) in B-mode **(B)** pseudoaneurysm in power-Doppler mode. **(C)** hematoma (white arrow) on computed tomography **(D)** pseudoaneurysm (white arrows) on the next caudal slice of the hematoma.

A 38-year-old primiparous pregnant Japanese woman was transferred to our hospital because of pregnancy-induced hypertension at 37 + 0 weeks’ gestation. Until that time, her pregnancy had been uneventful. On admission, the patient’s height was 156 cm, her body weight was 66 kg, and her blood pressure was 144/67 mmHg. Proteinuria was negative. The patient’s labor progressed rapidly on the day of admission, with full dilation of the cervix 8 hours after the onset of labor. Because of prolonged deceleration on cardiotocogram, vacuum extraction was performed when the fetal head reached the +2 station. The 2,405 g male neonate had an Apgar score of 8 at 1 min and 9 at 5 min after delivery. A vaginal laceration extending 5 cm from the introitus at the 6 o’clock position was repaired. Two hours after delivery, massive bleeding from the vaginal sutures was found. When all sutures were released, pulsatile bleeding was confirmed, necessitating a second repair.Two hours after the second repair, transvaginal ultrasonography showed a hyperechoic 18 × 20 mm mass at the 5 o’clock position on the vaginal wall, suggestive of a hematoma, within which a 6 × 7 mm anechoic mass with pulsation was described (Figure 1A). Power-Doppler ultrasonography showed blood flow signals in the anechoic mass (Figure 1B), and arterial waveforms were detected in pulsed-Doppler mode. Contrast-enhanced CT revealed an 8 mm diameter pooling of contrast adjacent to an 18 × 32 mm mass in the pelvis (Figure 1C, D).

**Figure 2:**
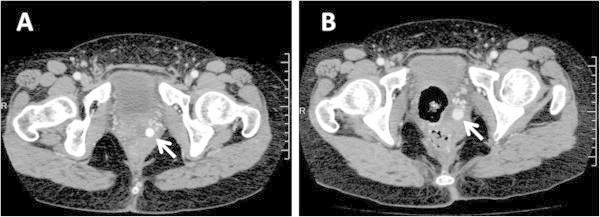
**Pelvic contrast-enhanced computed tomography pseudoaneurysms (white arrows). (A)** on the fourth postpartum day **(B)** on the sixth postpartum day.

**Figure 3:**
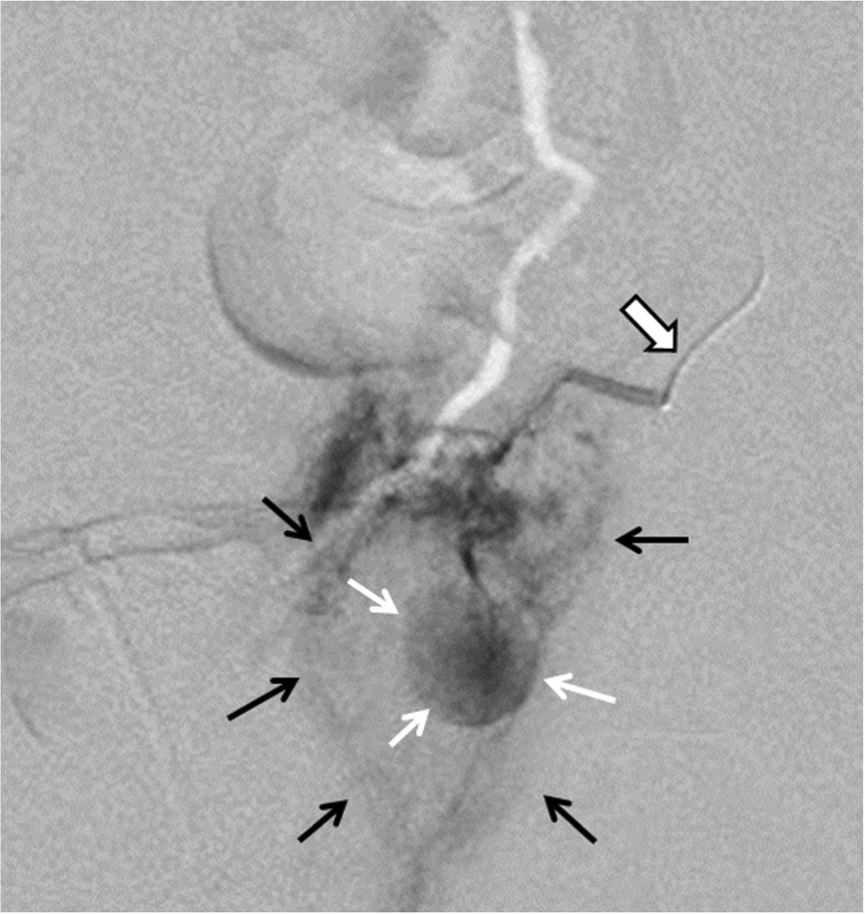
**Pelvic angiography on the sixth postpartum day showed strong staining (white arrows) with extravasation (black arrows) indicating ruptured pseudoaneurysm arising from the left vaginal artery (open arrow).**

By the fourth postpartum day, the pseudoaneurysm had grown to 10 mm in diameter (Figure 2A). Because of the risk of rupture, elective embolization was planned. On the sixth postpartum day, the patient experienced abdominal pain and massive vaginal bleeding of 260 ml. Contrast-enhanced CT showed that the pseudoaneurysm had grown to 14 mm (Figure 2B). Emergency selective angiography showed strong staining with extravasation from a peripheral branching vessel of the left vaginal artery, indicating ruptured pseudoaneurysm (Figure 3). Selective embolization was performed using n-butyl-2-cyanoacryate. No blood transfusion was necessary. The patient was discharged without further complications on the 13^th^ postpartum day.

## Discussion

Ours is the ninth reported case of vaginal pseudoaneurysm after delivery. Clinical characteristics of these cases are summarized in Table 1 (Matsuhashi et al. 2001; Soyer et al. 2008; Arab and Dy 2011; Nagayama et al. 2011). Rapid progression of labor with vacuum or forceps delivery is a probable risk factor for vaginal pseudoaneurysm. However, two of the nine reported cases had uncomplicated spontaneous delivery with no vaginal lacerations. All cases but ours were diagnosed by angiography and/or contrast-enhanced CT because of massive vaginal bleeding in the first ten postpartum days. As far as we know, our case is the first in which an unruptured vaginal pseudoaneurysm was diagnosed using ultrasonography. In our case, the vaginal laceration was surgically repaired twice, leading us to perform ultrasound examination after the second repair. This examination led to early detection of the unruptured pseudoaneurysm.

**Table 1:** **Summary of clinical characteristics of nine cases with vaginal pseudoaneurysm**

Case	Age (y)	Parity	Complication	Assisted delivery	Vaginal laceration	Diagnostic modality	Size (mm)	Onset of rupture	Feeding artery	Blood transfusion
1 (Zimon et al. 1999)	33	0	Hemophilia A	Spontaneous	-	Angiography	N/A	Day 16	Lt. internal pudendal	+
2 (Pelage et al. 1999)	34	1	None	Spontaneous	+	Angiography	6	Day 0	Lt. obturator	+
3 (Pelage et al. 1999)	28	2	None	Spontaneous	-	Angiography	6	Day 0	Lt. obturator	+
4 (Pelage et al. 1999)	39	1	None	Spontaneous	-	Angiography	10	Day 0	Rt. internal pudendal	+
5 (Pelage et al. 1999)	30	1	None	Forceps	+	Angiography	8	Day 0	Lt. obturator	+
6 (Pelage et al. 1999)	35	1	None	Forceps	-	Angiography	7	Day 10	Rt. uterine	+
7 (Padavala and Ahluwalia 2004)	29	1	None	Vacuum	+	Angiography	25	Day 2	Lt. internal pudendal	+
8 (Cooper et al. 2004)	27	0	None	Vacuum	+	CT	N/A	Day 4	Lt vaginal	+
9*	37	0	None	Vacuum	+	US/CT	14	Day 6	Lt vaginal	-

*, our case. CT, computed tomography, US, ultrasonography.

The differential diagnoses of pseudoaneurysm are arteriovenous malformations (AVMs), including arteriovenous fistula, and true aneurysm. Ultrasound diagnosis of pseudoaneurysm is based on the presence of one or two cystic masses with color- and/or power-Doppler flow signals and high-resistance arterial flow waveforms in pulsed-Doppler mode, often in association with a to-and-fro waveform pattern (Nagayama et al. 2011; Zimon et al. 1999; Wald 2003; McGonegle et al. 2006). AVMs have focally or asymmetrically distributed small anechoic spaces with a tangle of tortuous vessels showing multidirectional flow in color-Doppler mode, termed a two-color mosaic pattern (Kwon and Kim 2002; Cooper et al. 2004; Yi and Lee 2012). Pulsed-Doppler mode shows low-resistance arterial waveform patterns. This case had the typical ultrasonographic patterns of pseudoaneurysm. It is not possible to differentiate true aneurysm from pseudoaneurysm by color/pulsed-Doppler flow profile alone. However, the presence of a hematoma can aid the diagnosis of pseudoaneurysm. In addition, the size of a true aneurysm is not likely to change over the course of several days.

We did not initially perform embolization of the unruptured pseudoaneurysm in this case, because the patient did not have clinical signs, including bleeding from the vaginal laceration. Instead, we carefully monitored the size of the pseudoaneurysm using ultrasonography and contrast-enhanced CT. Sequential ultrasound examinations did not show growth, but contrast-enhanced CTs showed ongoing development of the pseudoaneurysm. Ultrasonography scans various sections of a targeted mass and an image of the maximal dimension can be viewed. In contrast, CT scans the targeted mass in 5-mm slices perpendicular to the body axis and the image can be viewed as an average within the sliced area. In theory, both contrast-enhanced CT and ultrasonography can detect changes in the size of pseudoaneurysms once they grow beyond the resolution. One possible reason that ultrasonography may fail to detect changes in pseudoaneurysm size is improper scanning.

The definitive diagnosis of pseudoaneurysm is made by angiography. However, ultrasonography is a non-invasive and clinically useful modality in the differential diagnosis of pseudoaneurysm. Contrast-enhanced CT with or without ultrasonography can be useful for sequential monitoring of the size of unruptured pseudoaneurysms.

The average size of vaginal pseudoaneurysms including our case is 11 mm, with a minimum size of 6 mm (Matsuhashi et al. 2001; Soyer et al. 2008; Arab and Dy 2011; Nagayama et al. 2011). In retrospect, we could have performed angiography for embolization when the unruptured pseudoaneurysm was diagnosed, or at the latest when ongoing development of the pseudoaneurysm was recognized, irrespective of whether symptoms were present. However, awareness of the diagnosis and immediate intervention for hemostasis after rupture enabled the patient to escape life-threatening blood loss and minimized complications.

Vaginal pseudoaneurysm is a rare complication after delivery but there may be unrecognized cases that spontaneously resolve. Routine transvaginal ultrasound examinations using B-mode may be clinically useful after severe vaginal laceration to rule out small and medium-sized hematomas, which may be missed on digital examination. In patients with severe vaginal laceration with pulsatile bleeding and in those with hematoma detected on routine examination, extensive ultrasonography with color flow profile may be necessary to detect vaginal pseudoaneurysm.

## Consent

Written informed consent was obtained from the patient for publication of this case report and any accompanying images.
